# 

**Published:** 1899-05

**Authors:** 


### CONTENTS.

ORIGINAL COMMUNICATIONS—

Speech of Hon. N. J. Hammond, LL.D. .......... 145
A Memorial Address on the Late Dr. Henry Campbell Doughty. By Joseph Eve Allen, Augusta, Ga. 159
Address of Welcome. By Hon. Emory Speer, Macon, Ga .......... 164

SOCIETY REPORTS—

Medical Association of Georgia .......... 169

EDITORIAL NOTES AND COMMENTS—

The Late Meeting of the Georgia Medical Association .......... 189
The Serum of Sobriety .......... 191
Isolation of Consumptives .......... 192
Death of Hon. N. J. Hammond .......... 193
Next Meeting of State Medical Examining Board .......... 194
Examination Questions Before the State Medical Examining Board .......... 195

CONTENTS CONTINUED .......... vi

